# Occurrence of uveitis in a population-based cohort of inflammatory bowel diseases followed for 10 years: an observational study

**DOI:** 10.1136/bmjophth-2023-001318

**Published:** 2023-07-14

**Authors:** Elisabet Granstam, Anders Rönnblom

**Affiliations:** 1 Center for Clinical Research Region Västmanland, Uppsala Universitet, Västerås, Sweden; 2 Ophthalmology, Region Västmanland, Västerås, Sweden; 3 Medical Sciences, Uppsala University Faculty of Medicine, Uppsala, Sweden

**Keywords:** inflammation, epidemiology, treatment medical

## Abstract

**Objective:**

The coexistence of inflammatory bowel diseases (IBDs) and uveitis has been known for 100 years. The reported frequency by which these conditions appear in the same patient has varied considerably. The aim of this study was to investigate the occurrence of uveitis in a well-defined population-based cohort of patients with IBD including all age groups and followed for at least 10 years.

**Method and analysis:**

All newly diagnosed patients with ulcerative colitis and Crohn’s disease in the county of Uppsala between 2005 and 2009 were prospectively followed. At the end of 2022, the medical notes were checked and all contacts with the healthcare system regarding ocular symptoms were scrutinised.

**Results:**

A total of 330 patients with ulcerative colitis and 153 patients with Crohn’s disease were included in the cohort. Four hundred and forty-two of these (91.5%) could be followed for 10 years or until death. Thirteen patients with ulcerative colitis were affected by uveitis (3.9%), and one of the patients with Crohn’s disease (0.7%). Most often the uveitis was diagnosed after the bowel disease (median 8.9 years, 7.7 years SD).

**Conclusion:**

Low occurrence of uveitis was identified in the IBD population. All affected individuals except one were diagnosed with ulcerative colitis. Most of the patients had their eye disease around 10 years later than their IBD diagnosis. It is suggested that systemic anti-inflammatory treatment for the IBD protects against intraocular inflammation in this cohort.

**WHAT IS ALREADY KNOWN ON THIS TOPIC**

- Uveitis is a known extraintestinal manifestation of inflammatory bowel disease (IBD).

**WHAT THIS STUDY ADDS**

- Low occurrence of uveitis was observed in a 10-year follow-up population-based cohort study of Swedish patients with IBD.
- Most of the patients had their eye disease around 10 years later than their IBD diagnosis.

**HOW THIS STUDY MIGHT AFFECT RESEARCH, PRACTICE OR POLICY**

- Uveitis is a late follower of IBD. Collaboration between internal medicine and ophthalmology is important to ensure adequate systemic anti-inflammatory treatment for these patients.

## Introduction

Inflammatory bowel disease (IBD) is the composite term for ulcerative colitis (UC), Crohn’s disease (CD) and IBD-unclassified. The incidence of these diseases has increased in many parts of the world during the second half of the 20th century.^1^ In the county of Uppsala, the incidence of UC increased from 2/100 000 inhabitants/year in 1945 to 20/100 000/year in 2009.^2 3^ The incidence of CD was 9.9/100 000/year during the most recent investigation^4^ whereas the incidence of IBD-unclassified has been reported as 5.2/1 00 000/year.^5^ IBDs are chronic relapsing diseases that can affect all age groups, but predominantly individuals in their thirties. A characteristic feature among patients with IBD is extraintestinal manifestations.^6^

Case reports describing an association between UC and CD and uveitis were published in 1925 and 1932, respectively,^7 8^ but more systematic descriptions were not done until the 60s and 70s.^9 10^

There is considerable variation in the reported frequency of intraocular inflammation among individuals suffering from IBD. A registry study from Canada in 2001 including patients with IBD for at least 10 years, found that 2% of patients with UC and 1.5% of the patients with CD had received a diagnosis of uveitis.^6^ On the other hand, a study of 1249 patients with IBD from Switzerland (2015) found uveitis in 13.7% of the patients.^11^ Ophthalmologically, IBD is a non-infectious disease known to be associated with uveitis.^12^

The aim of this study is to describe the occurrence of uveitis in a well-defined population-based cohort of patient diagnosed with IBD between 2005 and 2009 including all age groups and followed for at least 10 years.

## Patients and methods

This study derived its patients from the IBD Cohort Uppsala Region (ICURE cohort).^3 4^ The ICURE cohort recruited all patients with a new diagnosis of CD and UC in Uppsala county from 2005 to 2006 and expanded inclusion to the whole Uppsala region from 2007 to 2009. The patients included in this study were from the Uppsala health region and the recruitment was accomplished 2005–2009 in the area served by the University Hospital. All probable new cases of patients with IBD were identified through (A) gastroenterologists reporting to local investigator, (B) reviewing of all colonoscopy records at the department by local investigator and (C) reviewing lists of outpatient clinic visits. A few additional cases were identified through radiology or pathology rounds.

All included individuals were given an identification number. Only the responsible researchers had access to the code list. Analysis of data was performed deidentified.

Standard diagnostic methods were used and the patients with IBD were classified according to the Montreal classification.^13^ The Montreal classification describes the extent and behaviour of CD and includes a classification system for UC. It is widely used in both clinical practice and in research.

Classification of uveitis followed the Standardizaton of uveitis nomenclature (SUN) classification.^14^ Uveitis is classified based on the primary anatomic site in which intraocular inflammation is detected. In anterior uveitis, the primary site of inflammation is the anterior chamber including the iris and ciliary body, in intermediate uveitis the primary site of inflammation is the vitreous body whereas in posterior uveitis it is the retina or choroid. In panuveitis, all parts of the uvea are affected by inflammation.^14^ Identified episodes of uveitis were assessed as mild, moderate or severe based on clinical findings and disease course as reported by the ophthalmologist in the patient ophthalmology charts.

During the period with patient recruitment, Uppsala county encompassed on average 330 000 inhabitants. During 2022, the medical records of the patients included in the study were analysed and all contacts with the department of ophthalmology were further scrutinised. This study included all recorded contacts in the medical notes as early as possible also before the diagnosis of IBD.

This IBD-cohort has previously been studied with respect to the occurrence of psoriasis and hepatobiliary diseases, and results from these studies will be presented in the discussion.

### Statistics

All data analyses were performed using the software STATISTICA (V.10; 2011; StatSoft, Tulsa, OK; http://www.statsoft.com). Non-parametric continuous variables are presented as means and medians and were tested for significance with the Mann-Whitney test whereas categorical variables were tested with the χ^2^ test. Fisher’s exact test was used when small numbers. A p<0.05 was considered significant.

## Results

In total, 483 patients with IBD were included in the study, of which 330 patients had UC and 153 patients with CD. In all, 56 individuals (12%) were migrant subjects, none of whom were affected by uveitis. The median follow-up was 11.3 years (IQR=10.3–12.7) and 442 of the patients (91.5%) could be followed for 10 years or until death. Clinical characteristics of the patients are presented in table 1. The patients with CD were more exposed to all sorts of drugs compared with patients with UC, except for mesalamine (5-aminosalicylic acid). Biologics used were infliximab, adalimumab and vedolizumab.

**Table 1:** Clinical characteristics of the cohort

	Ulcerative colitis (UC), all	UC, no uveitis	UC with uveitis	P value	Crohn’s disease (CD), all	CD, no uveitis	CD with uveitis	P value
N (%)	330	317	13 (3.9)		153	152	1 (0.7)	
Age md	33.5	33	39	0.28	28.0	27.5	41	
range	3–86	3–86	17–74		4–78	4–78		
Age uveitis md			52				34	
Gender m/f	174/156	167/150	7/6		75/78	75/77	0/1	
Extent*				0.053				
E1	108	103	5					
E2	109	108	1					
E3	89	82	7					
Unknown	24	24	0					
Location†								
L1					33	33	0	
L2					89	89	0	
L3					31	30	1	
L4					31	31	0	
Behaviour‡								
B1					119	118	1	
B2					19	19	0	
B3					15	15	0	
P					16	16	0	
Smoking				0.33§				
Never	174	168	6		85	84	1	
Active	40	37	3		34	34	0	
Former	103	100	3		24	24	0	
Missing data	13				10	10	0	
Drugs								
5-ASA**	316 (96%)	304	12	NS	126 (82%)			<0.01¶
IMM††	109 (33%)	107	2	NS	101 (66%)			<0.01
Steroids	228 (69%)	219	9	NS	128 (84%)			<0.01
biologics	35 (11%)	35	0	NS	37 (24%)			<0.01
SASP‡‡	51 (15%)	49	2	NS	53 (35%)			<0.01

*E1=rectal inflammation, E2=left-sided colitis, E3=inflammation above splenic flexure.

†L1=inflammation in distal ileum, L2=inflammation in colon, L3=inflammation in distal ileum and colon, L4=inflammation in proximal ileum.

‡B1=inflammation without complication, B2=stenosis, B3=bowel penetration, p=perianal disease.

§χ2 test, comparison iritis versus no iritis.

¶χ2 test, comparison ulcerative colitis versus Crohn’s disease.

**5-ASA, aminosalicylic acid, mesalamine.

††Immunomodulator (azathioprine, 6-mercaptopurin).

‡‡Sulfasalazine.

NS, not statistically significant.

**Figure 1:**
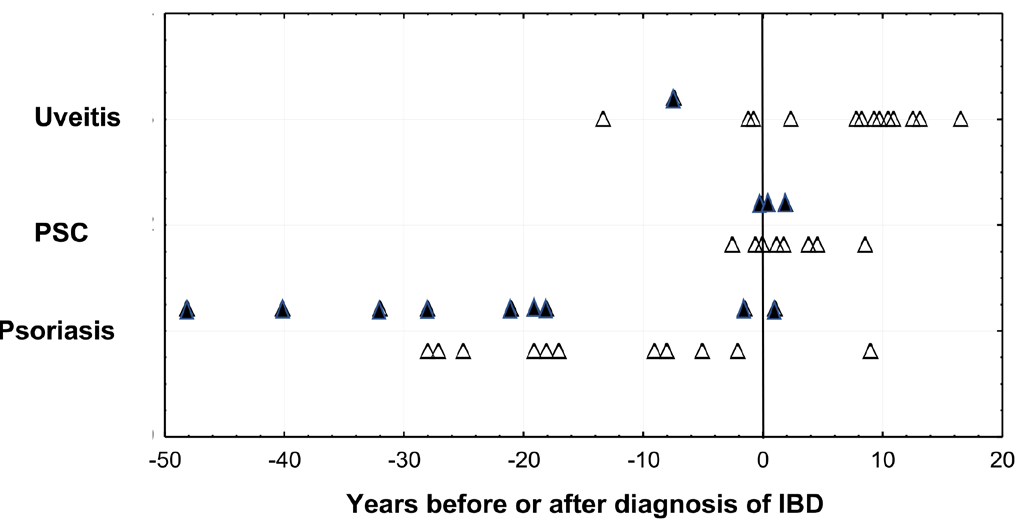
Diagnosis of extraintestinal manifestations of IBD in relation to diagnosis of IBD. Vertical line denotes time for IBD diagnosis. Open markers denote ulcerative colitis, black markers denote Crohn’s disease. Data for PSC and psoriasis from the same IBD cohort is included for comparison. IBD, inflammatory bowel disease; PSC, primary sclerosing cholangitis.

Fourteen patients were diagnosed with uveitis during the observation period, 13 with UC (13/330, 3.9%) and 1 with CD (0.7%). The gender distribution among patients with intraocular inflammation was similar as compared with the whole IBD group. Ten out of 14 patients experienced an episode of uveitis after contracting IBD, whereas 4 patients experienced uveitis before the diagnosis of IBD had been established. One patient was diagnosed with anterior uveitis 13 years before the UC was diagnosed and two other patients suffered from anterior uveitis 1 year before the bowel disease was diagnosed. The sole patient with CD was affected by anterior uveitis 17 years before the bowel disease. Most of the patients had their eye disease around 10 years after contracting IBD (figure 1).

One individual experienced an episode of intraocular inflammation concomitantly with active inflammation of the bowel and two patients had recently stopped taking drugs (azathioprine and sulfasalazine, respectively) for the bowel before the intraocular inflammation appeared. Half of the patients in clinical remission with regard to their bowel disease were off systemic anti-inflammatory therapy.

Median age at the time of uveitis was 52 years (SD 18.0, range 23–84). Both eyes were sequentially affected in seven patients. Six patients experienced recurrent disease with at least one additional episode of uveitis during follow-up. All episodes were sudden in onset, limited in duration and all but one episode was restricted to the anterior chamber. In this case, cells in the vitreous body were observed, a sign of intermediate uveitis and posterior segment involvement. Increased intraocular pressure and corneal oedema were also present. No findings of choroidal or retinal involvement or macular oedema were noted in any patient. All patients received topical anti-inflammatory treatment with steroids and cycloplegic eye-drops. There were two patients in need of systemic anti-inflammatory therapy. In patient 6, sulfasalazine, which recently had been stopped, was reintroduced by the ophthalmologist. Patient 10 was affected by uveitis before IBD was diagnosed and was the only one who was prescribed systemic steroids for the uveitis (table 2).

**Table 2:** Ocular findings, patients with inflammatory bowel disease and uveitis

No	Bilat	Severity of intraocular inflammation	Additional findings in anterior segment	Posterior segment inflammation involvement	Topical therapy	Systemic therapy	Recurrent disease	Normal VA
1	No	Moderate			Yes	No	No	Yes
2	No	Moderate			Yes	No	No	Yes
3	No	Moderate			Yes	No	No	Yes
4	No	Mild			Yes	No	No	Yes
5*	No	Moderate			Yes	No	No	No
6	No	Severe	Increased IOP, corneal oedema	Yes intermediate uveitis	Yes	Yes†	No	No
7	Yes	Severe			Yes	No	No	Yes
8	Yes	Moderate			Yes	No	No	Yes
9	Yes	Mild			Yes	No	Yes	Yes
10	Yes	Moderate			Yes	Yes	Yes	Yes
11	No	Severe			Yes	No	Yes	No
12	Yes	Moderate			Yes	No	Yes	Yes
13	Yes	Mild			Yes	No	Yes	Yes
14	Yes	Moderate			Yes	No	Yes	Yes

Patient number 10 had CD.

*This patient (5) had a history of previous treatment for choroidal malignant melanoma.

†Reintroduction of sulfasalazine.

CD, Crohn’s disease; IOP, Intraocular pressure.

## Discussion

In this population-based IBD cohort, a low occurrence of uveitis was observed. All episodes of intraocular inflammation affected patients diagnosed with UC (3.9%) except for one individual with CD (0.7%). Most of the patients had their eye disease around 10 years after their IBD diagnosis.

Our results are in line with the Canadian population-based registry study limited to patients with an IBD diagnosis for at least 10 years and including 4454 patients, which found a prevalence of 2% and 1.5% for UC and CD, respectively.^6^ Our findings also adhere to results from a population-based Swedish study from 1990 among 1274 patients with UC which found 20 patients with anterior uveitis (1.6%).^15^ Three studies from the neighbour country Denmark report a prevalence of uveitis in UC between 0.9% and 1.5% and in the interval 0.1%–2.6% in CD.^16–18^ In contrast, a higher prevalence of uveitis was reported in a Swiss study,^11^ probably explained by the selective recruitment of patients included in the evaluated cohort.

The Norwegian IBSEN study is a population-based IBD study that resembles our study in many respects, except for starting 14 years earlier and with less prevalent use of immunomodulatory drugs and biologics.^19^ After 5 years of follow-up, a prevalence of anterior uveitis of 2.1% and 3.1% was found for UC and CD, respectively. In contrast to these findings, we observed only one patient with CD and intraocular inflammation, and our patients with UC were affected later in the disease course. If our study had terminated after 5 years, only 1.2% of patients with UC would have been diagnosed with uveitis.

A recent meta-analysis has reported that patients with CD has an approximately 1.6-fold significantly increased odds of developing uveitis compared with UC.^20^ No such increased risk for patients with CD was observed in our study. The meta-analysis generally reported lower prevalence of uveitis among the UC-patients. The findings were based mainly on registry studies. Also, patients under the age of 18 were excluded.^20^

We hypothesise that the low occurrence of uveitis among patients with CD in comparison with those suffering from UC in our cohort might be a consequence of a more active pharmacological treatment among patients with CD. All drugs that were more frequently used among patients with CD (ie, steroids, sulfasalazine, antimetabolites and biologics) have been demonstrated to have a positive clinical effect on intraocular inflammation.^21 22^ Two of the patients in the cohort developed uveitis after withdrawal of azathioprine and sulfasalazine, respectively, which further supports a relation between drug use and development of uveitis.

Treatment of IBD has changed considerably during recent years with more frequent use of classical immunomodulatory drugs such as azathoprine and the introduction of biologicals, that is, anti-TNF drugs.^23^ At the same time, the frequency of surgery for IBD has decreased and is reserved for the most severe cases.^24^ We speculate that the later appearance of uveitis in our IBD cohort and the overall low occurrence rate of intraocular inflammation could be a consequence of a widespread use of azathioprine and biologicals.

In our study, we found that only two of the patients were prescribed immunomodulatory treatment for their intraocular inflammation by their ophthalmologist. In the international expert-led consensus initiative fundamentals of care of uveitis (FOCUS) the use of non-corticoid systemic immunomodulatory therapy in non-infectious uveitis is encouraged.^25^ A closer collaboration between internal medicine and ophthalmology is important to ensure adequate systemic anti-inflammatory treatment for these patients.

The episodes of intraocular inflammation identified in our cohort were sudden in onset and limited in duration and half of them were unilateral. All episodes were restricted to the anterior chamber except for one episode of intermediate uveitis. Only 6 out of 14 patients had recurrent disease with at least two documented episodes of intraocular inflammation. Different clinical characteristics of uveitis associated with different simultaneous inflammatory disease have been reported. Uveitis in patients with psoriatic arthritis is more likely to be insidious in onset, continuous in course and active bilaterally.^26^ Uveitis is particularly common among patients with juvenile idiopathic arthritis (JIR), and in a recent population-based Nordic cohort, 22.1% of the individuals were affected with ocular inflammation.^27^ Uveitis in JIR is often clinically silent, and therefore, screening programmes for children with JIR have been designed.^28^,

We have earlier reported the correlation between IBD and hepatobiliary diseases and psoriasis.^29 30^ There is an obvious difference regarding the timing between these concomitant diseases. While psoriasis usually presents many years before IBD,^29^ and hepatobiliary diseases, particularly primary sclerosing cholangitis (PSC) at the same time as IBD,^30^ this study demonstrates that uveitis is a late follower of IBD. The reason for this could be at least twofold. The genetic impact on psoriasis is greater than on IBD,^31^ thus possibly making the skin disease manifest earlier in vulnerable individuals. Furthermore, a pathological process in the skin is observable earlier than in the bowel. PSC can have a long preclinical phase, but liver function is regularly tested in the follow-up of IBD, thereby identifying the disease early. A case–control study of 124 patients with IBD with ocular manifestations (uveitis, episcleritis or scleritis) compared with 3328 patients with IBD without ocular manifestations found associations with other extraintestinal manifestations (erythema nodosum and arthritis) but only nominal association with single nucleotide polymorphisms.^32^

A limitation of this study is the retrospective collection of ophthalmological data from clinical patient charts. The inclusion of patients with the underlying bowel disease was, however, done prospectively and the target population includes all age groups, and the majority has been followed for 10 years or until death. Thus, we conclude that the study has a high external validity.

In summary, low occurrence of uveitis was identified in the IBD population. All affected individuals except one were diagnosed with UC. The majority of the patients had their eye disease around 10 years after their IBD diagnosis. It is hypothesised that systemic anti-inflammatory treatment for the IBD protects against intraocular inflammation in this cohort.

## Data Availability

Data are available on reasonable request. Data are available on reasonable request. The datasets used and analysed during the current study are available from the corresponding authors on reasonable request.
